# Sex differences in cerebral pulsatility and damping: A 4D flow MRI study

**DOI:** 10.1113/EP092630

**Published:** 2025-07-19

**Authors:** Sarean Harmoni A. Gaynor‐Metzinger, Alexander M. Norby, Brandon G. Fico, M. Erin Moir, Nicole A. Loggie, Kathleen B. Miller, Adam T. Corkery, Andrew G. Pearson, Anna J. Howery, Leonardo A. Rivera‐Rivera, Kevin M. Johnson, Sterling C. Johnson, Oliver Wieben, Ryan D. Zea, Jill N. Barnes

**Affiliations:** ^1^ Bruno Balke Biodynamics Laboratory, Department of Kinesiology University of Wisconsin‐Madison Madison Wisconsin USA; ^2^ Department of Exercise Science and Health Promotion Florida Atlantic University Boca Raton Florida USA; ^3^ Department of Health and Exercise Science, Morrison Family College of Health University of St. Thomas St. Paul Minnesota USA; ^4^ Department of Medicine, School of Medicine and Public Health University of Wisconsin‐Madison Madison Wisconsin USA; ^5^ Wisconsin Alzheimer's Disease Research Center, School of Medicine and Public Health University of Wisconsin‐Madison Madison Wisconsin USA; ^6^ Department of Medical Physics, School of Medicine and Public Health University of Wisconsin‐Madison Madison Wisconsin USA; ^7^ Department of Radiology, School of Medicine and Public Health University of Wisconsin‐Madison Madison Wisconsin USA

**Keywords:** ageing, basilar artery, cerebral blood flow, haemodynamics, internal carotid artery, magnetic resonance, middle cerebral artery, vascular physiology

## Abstract

Cerebral pulsatility is a potential marker of cerebrovascular health, yet little is understood about sex differences in cerebral pulsatility with age, especially within different cerebral arteries. Additionally, cerebral damping can blunt cerebral pulsatility and might decline with age. Therefore, we aimed to identify sex differences in cerebral pulsatility and damping across the adult lifespan. Forty‐three young, 67 middle‐aged and 54 older adults had cerebral haemodynamics measured in the internal carotid arteries (ICAs), middle cerebral arteries (MCAs) and basilar artery using 4D flow MRI. Cerebral pulsatility index (PI) and damping factor (DF) were calculated. Young females had lower PI than young males in the ICAs (*p <* 0.05 for both), and middle‐aged females had lower PI than middle‐aged males in the right ICA (*p <* 0.01). In contrast, older females had greater PI than older males in the right ICA (*p <* 0.01) and in the right MCA (*p <* 0.05). Only the DF between the right ICA and MCA was lower in young females than in young males (*p <* 0.001). Taken together, females experience greater age‐related elevations in cerebral pulsatility in comparison to males, especially within the proximal arteries of the anterior circulation. Damping was not different between males and females within the proximal arteries of anterior circulation, suggesting a different underlying mechanism. Overall, our findings suggest sex‐specific trends in cerebral pulsatility with age, although the mechanisms driving this require further exploration.

## Abstract (highlights)

- **What is the central question of this study?**
  Are there sex differences in cerebral pulsatility and damping across the adult lifespan?
- **What is the main finding and its importance?**
  Females had greater age‐related increases in cerebral pulsatility than males, beginning with lower cerebral pulsatility at a young age and having higher cerebral pulsatility by older age, particularly within the proximal arteries in the anterior circulation. There were no sex differences in damping within the proximal arteries in the anterior circulation, suggesting that sex‐specific trends in cerebral pulsatility with age might be attributable to a different underlying mechanism.

## 1 INTRODUCTION

Highly perfused organs, such as the brain, are sensitive to perturbations in blood flow (De Roos et al., 2017). To reduce such perturbations, the large elastic extracranial arteries (e.g., aorta and carotid arteries) dampen the pulsatile flow waves created by ventricular ejection. This damping by the large extracranial arteries promotes more continuous flow through the systemic circulation, including the brain (Belz, 1995). Inadequate or impaired damping of pulsatile flow could lead to greater transmission of pulsatile energy downstream, thereby exposing high‐flow end organs, such as the brain, to greater flow pulsatility. Recently, our group demonstrated that stiffness in the large elastic extracranial arteries, which presumably reduces the ability to dampen pulsatile flow, was positively associated with greater cerebral pulsatility in the large intracranial arteries (Fico et al., 2022). Furthermore, greater cerebral pulsatility has emerged as a likely contributor to cerebral microvascular damage and subsequent structural damage within the brain (e.g. white matter hyperintensities) (Bateman, 2004; De Roos et al., 2017; Scuteri et al., 2011). Along these lines, previous work has demonstrated that elevated flow pulsatility is associated with total brain atrophy (Mitchell et al., 2011). Thus, these factors might augment the risk of developing cerebrovascular pathologies, mild cognitive impairment and dementia (Bateman, 2004; Bateman et al., 2008; Zlokovic et al., 2020).

Postmenopausal females have a greater risk for age‐related cognitive impairment and development of several neurodegenerative diseases compared with males of a similar age, which might be attributable, in part, to disproportionate increases in cerebral pulsatility (Levine et al., 2021; Seshadri & Wolf, 2007). Previous work from our recent multi‐site study showed that young females had lower middle cerebral artery (MCA) blood velocity‐based pulsatility compared with young males (Alwatban et al., 2021). In addition, females had a greater rate of increase in pulsatility with advancing age compared with males, and the greatest rate of increase occurred around the age of menopause (Alwatban et al., 2021). This suggests that, relative to males, females might experience a greater magnitude of increase in cerebral pulsatility over the lifespan, even with lower cerebral pulsatility at a younger age. However, our previous study did not control for menopause status or use of menopausal hormone therapies, which could confound the effect of ageing on cerebral pulsatility in females.

Previous studies have often used transcranial Doppler ultrasound (TCD) to measure beat‐to‐beat cerebral pulsatility, typically within the MCA, which relies on blood velocity (Purkayastha & Sorond, 2013). However, TCD does not capture potential artery‐specific differences in cerebral pulsatility, and TCD provides pulsatility based on velocity and not flow. To address this limitation, we used four‐dimentional (4D) flow MRI for simultaneous blood flow acquisition in multiple intracranial arteries to provide a more comprehensive analysis of artery‐specific flow pulsatility (Markl et al., 2012; Miller et al., 2019; Rivera‐Rivera et al., 2016). We previously reported that 4D flow MRI is more sensitive to age‐related changes in cerebral haemodynamics when compared with TCD (Fico et al., 2023). Thus, using 4D flow MRI might provide a more thorough understanding of age and sex differences in cerebral pulsatility measures. In addition, by capturing cerebral pulsatility and damping within both left and right regions of the intracranial arteries, we can isolate region‐specific sex differences that may indicate unique age‐related deleterious changes in the cerebral vasculature (Luo et al., 2011). Furthermore, differences in the proximal arteries, such as the internal carotid arteries (ICAs). might be observed without detectable differences in the MCAs. Indeed, damping of the pulsatile flow has been shown to occur within specific arteries or regions along an artery (Van Tuijl et al., 2020), because the ICAs have a unique role in damping pulsatile flow attributable, in part, to their tortuous structure (Schubert et al., 2011; Van Tuijl et al., 2020).

Therefore, the purpose of this study was to examine the effect of sex in the age‐related differences in cerebral pulsatility and cerebral damping using 4D flow MRI to capture artery‐specific variables. We hypothesized that young females would have lower cerebral pulsatility in comparison to age‐matched males, and that this difference would diminish during middle‐age. We also hypothesized that older females would have greater cerebral pulsatility compared with age‐matched males, especially within proximal feed arteries (ICAs and basilar artery). Our exploratory hypothesis was that sex would also influence the age‐related differences in damping of the pulsatile flow either within the ICA or between the ICA and MCA.

## 2 MATERIALS AND METHODS

### 2.1 Ethical approval

All study protocols were approved via the Institutional Review Board of the University of Wisconsin‐Madison (2015‐1611, approval date 19 January 2017; 2016‐0403, approval date 28 September 2017; and 2020‐0108, approval date 7 January 2020) and performed in accordance with the *Declaration of Helsinki* (except for registration in a database, because these studies were not clinical trials). Written informed consent was received before participation. Data from these studies are used in this retrospective analysis.

### 2.2 Participant characteristics

One hundred and sixty‐four healthy and cognitively unimpaired adults (age range 20–89 years old) were included for this retrospective analysis and stratified by age into one of three groups: young (20–35 years; 25 males and 19 females), middle‐aged (45–65 years; 44 males and 23 females) or older (70–89 years; 19 males and 35 females). Participants were recruited for previous studies conducted within the Bruno Balke Biodynamics Laboratory (*n* = 71) (Corkery et al., 2021; Fico et al., 2022, 2023; Miller et al., 2018, 2019, 2021) or for the Wisconsin Alzheimer's Disease Research Center (ADRC) Clinical Core (*n* = 95) (Blazel et al., 2020). The experimental details pertaining to the previous studies can be found elsewhere. All participants in this retrospective analysis had a body mass index of <30 kg/m^2^, were not current smokers and were excluded if they met the following exclusion criteria: (1) diagnosed with hypertension and/or currently taking anti‐hypertensive medication; (2) had a history of stroke, diabetes or cardiovascular, renal/hepatic, haematological, peripheral vascular or neurovascular disease; or (3) had contraindications for MRI scans (determined using a health history questionnaire and MRI screening form) (Whelton et al., 2018). All young female participants were premenopausal and studied in the early follicular phase (days 2–6) of their menstrual cycle to minimize the effects of sex hormones. All middle‐aged and older females were postmenopausal for ≥1 year. Menopausal status was determined via self‐report using standardized health screening questionnaires.

### 2.3 Laboratory procedures

Participants attended two separate visits, for which they were requested to arrive fasted and having abstained from alcohol and caffeine for ≥4 h prior to each visit, which was confirmed upon arrival via self‐report. During the first visit, participants arrived at the laboratory, where they completed a health history questionnaire followed by anthropometric and vital measurements. These included height and weight, resting blood pressure (BP) with an automated BP cuff, and heart rate (HR) using either an automated BP cuff or a three‐lead ECG. During the second visit, participants arrived at the Wisconsin Institute for Medical Research (WIMR), where the MRI scan was conducted.

### 2.4 Magnetic resonance imaging procedures

The MRI scan was performed using a 3 T clinical MRI system (MR750, GE Healthcare, Waukesha, WI, USA) and an 8‐channel (*n* = 63) or 32‐channel (*n* = 103) head coil (Nova Medical Head Coil, Nova Medical, Wilmington, MA, USA). 4D flow phase contrast (PC) MRI was acquired for volumetric, time‐resolved PC MRI data with three‐directional velocity encoding (4D flow). To enable imaging in practical imaging times, a variant of 4D flow with 3D radial under sampling was used (PC VIPR), as previously described (Gu et al., 2005; Johnson et al., 2008). The imaging parameters were as follows: velocity encoding (Venc) = 80 cm/s, field of view = 220 mm, acquired isotropic spatial resolution = 0.7 mm × 0.7 mm × 0.7 mm, repetition time (TR) = 7.4 ms, echo time (TE) = 2.7 ms, flip angle = 8° or 10°, bandwidth = 83.3 kHz, 14 000 projection angles and scan time of ∼7 min. Time‐resolved velocity and magnitude data were reconstructed offline by retrospectively gating into 20 cardiac phases using temporal interpolation (Jing Liu et al., 2006; Rivera‐Rivera et al., 2016).

### 2.5 Flow analysis

The 4D flow MRI scans were evaluated offline, where investigators were blinded from the sex and age of the participants. The scans underwent background phase offset correction, eddy current correction (Schrauben et al., 2015) and automatic phase unwrapping to minimize the potential for velocity aliasing (Loecher et al., 2016). Artery segmentation of left and right ICAs, MCAs and basilar artery were performed in MATLAB using one of two in‐house tools, as previously described for semi‐ to full‐automated cerebrovascular haemodynamic analysis (Roberts et al., 2023; Schrauben et al., 2015). The ICAs were measured along the cervical and cavernous segments. The basilar artery was measured above the bifurcation of the vertebral arteries and below the superior cerebellar artery. The MCAs were measured at the M1 segment. Analysis of generated 2D cinematic image series with through‐plane velocities derived from 4D flow MRI data was conducted using a customized MATLAB tool (Roberts et al., 2023; Schrauben et al., 2015) that automatically detects the edge of the wall of each vessel. Diameter, cross‐sectional area and mean blood flow were then calculated for all vessel segments.

### 2.6 Cerebral pulsatility and damping factor analysis

Cerebral pulsatility for each artery was assessed via Gosling's pulsatility index (PI), which is calculated as (maximum flow − minimum flow)/mean flow (Gosling & King, 1974). The PI was calculated for the cervical and cavernous portions of the ICAs, the basilar artery and the MCAs. Cervical and cavernous portions of both ICAs were averaged prior to comparisons across age groups and between males and females. Cerebral pulsatile damping was measured using Gosling's damping factor (DF), which is calculated as proximal artery PI/distal artery PI (Gosling & King, 1974; Zarrinkoob et al., 2016). Two different DFs were calculated: (1) within‐ICA, which was between the cervical portion of the ICA and the cavernous portion of the ICA; and (2) ICA–MCA, which was the average between the cervical and cavernous regions of the ICA and the MCA.

### 2.7 Statistical analysis

Data analyses were performed using SPSS v.29 (SPSS, IBM Corp., Armonk, NY, USA). Data were visually inspected for violations of normality in tandem with Shapiro–Wilks tests prior to statistical analysis. Sex differences in participant characteristics were assessed using Welch's *t*‐tests or Mann–Whitney *U*‐tests within age groups, and sex differences were also assessed with age groups collapsed and can be found in the Supplemental file. Sex differences in PI and DF within age groups were evaluated using Welch's *t*‐tests or Mann–Whitney *U*‐tests. To investigate PI and DF further in males and females with age, general linear models or generalized linear models with a gamma distribution were performed, with sex as a fixed factor and age (continuous) as a covariate to evaluate the effects of sex and age on PI and DF within each artery of interest. Additional analysis was performed using pulse pressure (PP), which could contribute to pulsatility, as a covariate, and these results can be found in the Supplemental file. Statistical significance was established a priori at *p <* 0.05. Data are expressed as means (SD) unless otherwise stated.

## 3 RESULTS

### 3.1 Participant characteristics

Participant characteristics are presented in Table 1. A total of 164 adults (87 males and 77 females) were included in the cerebral pulsatility analysis. Haemodynamic data isolating the cervical and cavernous segments of the ICA for 3 participants (one young female, one young male and one middle‐aged male) could not be delineated owing to technical limitations; therefore, a total of 161 adults (85 males and 76 females) were included in the cerebral damping analysis. There were no differences in age (young, *p =* 0.370; middle‐aged, *p =* 0.176; older, *p =* 0.390), years of education (young, *p =* 0.189; middle‐aged, *p =* 0.162; older, *p =* 0.062) and HR (young, *p =* 0.231; middle‐aged, *p =* 0.099; older, *p =* 0.362) between males and females. Height and weight were greater in males compared with females in young (*p <* 0.001 for both), middle‐aged (*p <* 0.001 for both) and older (*P <* 0.001 for both) groups (Table 1). Systolic BP was higher in young and middle‐aged males compared with young and middle‐aged females (young, *p =* 0.010; middle‐aged, *p =* 0.002). Diastolic BP and mean arterial pressure were higher in middle‐aged males compared with middle‐aged females (*p <* 0.001 for both). The PP was also greater in young males compared with young females (*p =* 0.006) (Table 1).

**TABLE 1:** Participant characteristics.

Variable	Young (20–35 years)		*p*‐Value	Middle‐aged (45–65 years)		*p*‐Value	Older (70–89 years)		*p*‐Value
	Male	Female		Male	Female		Male	Female	
*n*	24	19		44	23		19	35	
Age, years	25 (4)	26 (4)	0.370	57 (4)	59 (4)	0.176	76 (4)	75 (5)	0.390
Education, years	18 (2)	17 (2)	0.189	18 (2)	17 (2)	0.162	17 (3)	16 (2)	0.062
Height, cm	178.1 (4.9)	168.2 (6.2)	**<0.001**	179.9 (7.5)	163.0 (6.7)	**<0.001**	176.6 (3.9)	160.7 (5.4)	**<0.001**
Weight, kg	75.3 (6.5)	64.7 (8.0)	**<0.001**	83.2 (11.1)	61.2 (9.9)	**<0.001**	79.5 (9.7)	64.1 (8.4)	**<0.001**
SBP, mmHg	123 (10)	116 (8)	**0.010**	126 (14)	114 (14)	**0.002**	125 (12)	131 (23)	0.220
DBP, mmHg	70 (6)	69 (7)	0.601	79 (8)	71 (8)	**<0.001**	75 (6)	76 (7)	0.557
MAP, mmHg	88 (6)	85 (6)	0.123	95 (10)	85 (10)	**<0.001**	92 (6)	94 (11)	0.262
PP, mmHg	54 (7)	47 (7)	**0.006**	46 (8)	43 (10)	0.135	50 (13)	55 (19)	0.262
HR, beats/min	54 (9)	58 (9)	0.231	58 (8)	62 (9)	0.099	62 (12)	65 (9)	0.362

*Note*: Data are presented as the mean (SD). The *p*‐values indicate differences between males and females within each age group. Values of *p <* 0.05 are in bold. Abbreviations: DBP, diastolic blood pressure; HR, heart rate; MAP, mean arterial pressure; PP, pulse pressure; SBP, systolic blood pressure.

### 3.2 ICA pulsatility index

**FIGURE 1:**
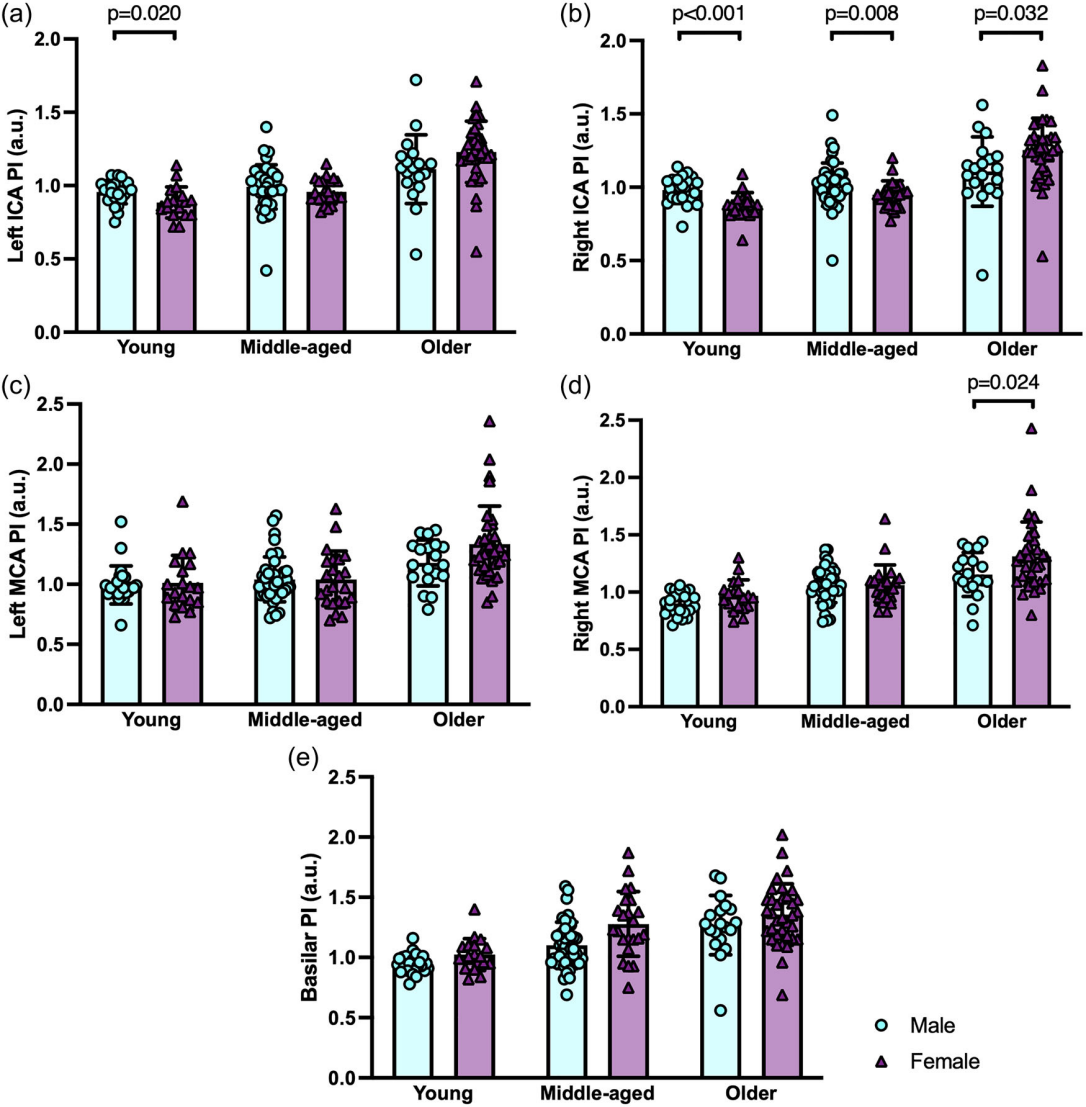
Sex differences in pulsatility index (PI) for males (turquoise, circles) and females (plum, triangles) in young (20–35 years; 25 males and 19 females), middle‐aged (45–65 years; 44 males and 23 females) and older (70–89 years; 19 males and 35 females) age groups are shown. Data are presented using bar graphs, with error bars indicating SD and overlapping data points shown. (a) Left internal carotid artery (ICA) PI (sex differences within: young, *p =* 0.020; middle‐aged, *p =* 0.119; and older, *p =* 0.079). (b) Right ICA PI (sex differences within: young, *p* **≤** 0.001; middle‐aged, *p =* 0.008; and older, *p* = 0.032). (c) Left middle cerebral artery (MCA) PI (sex differences within: young, *p* = 0.750; middle‐aged, *p* = 0.776; and older, *p* = 0.119). (d) Right MCA PI (sex differences within: young, *p* = 0.178; middle‐aged, *p* = 0.526; and older, *p* = 0.024). (e) Basilar artery PI (sex differences within: young, *p* = 0.690; middle‐aged, *p* = 0.089; and older, *p* = 0.228). Differences in PI between males and females within each age group were evaluated using Welch's *t*‐tests or Mann–Whitney *U*‐tests, with significance at *p <* 0.05.

**FIGURE 2:**
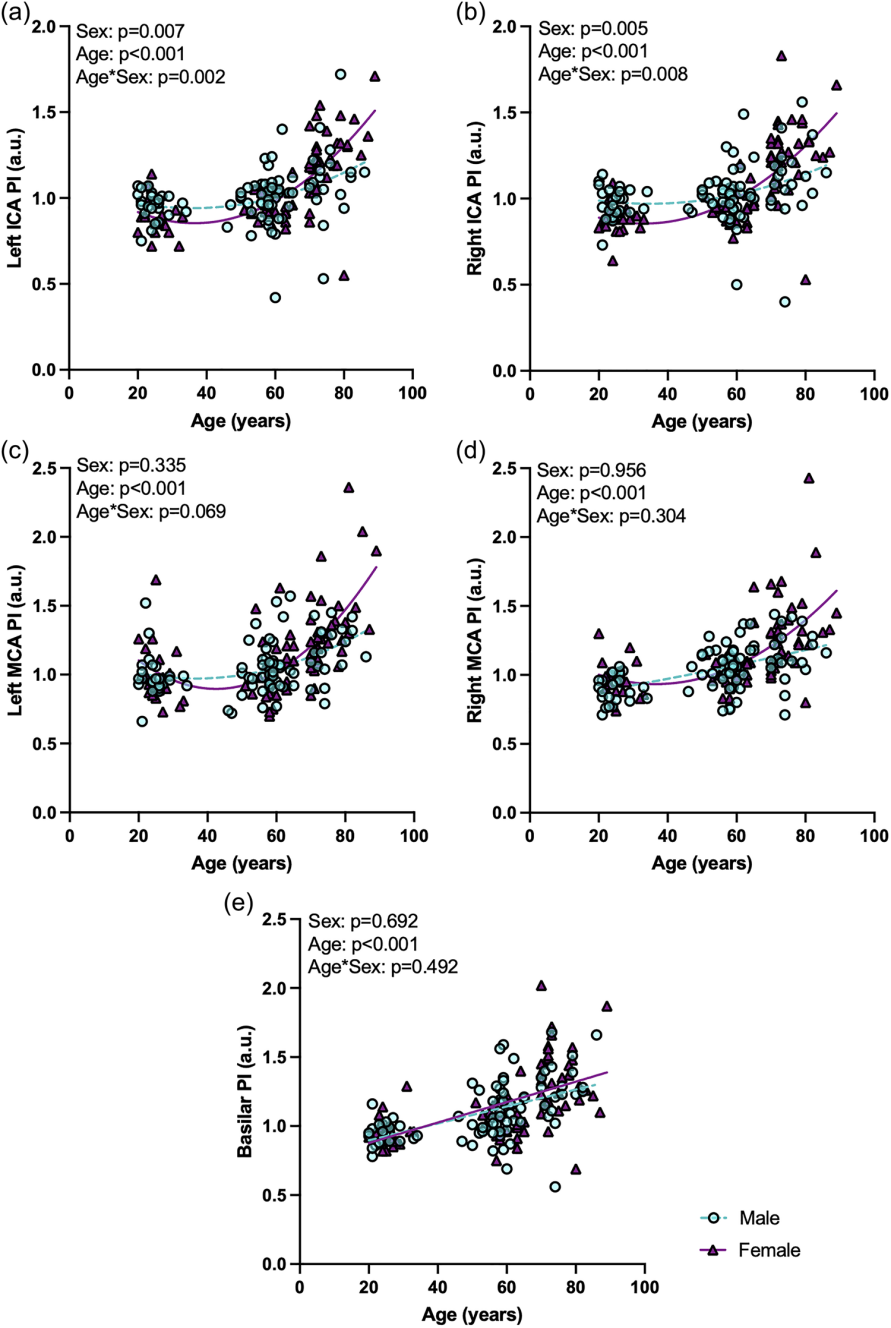
Pulsatility index (PI) with continuous age and sex was investigated using general linear models or generalized linear models with a gamma distribution as needed in males (*n* = 88; turquoise, circles) and females (*n* = 77; plum, triangles). (a) Left internal carotid artery (ICA) PI (interaction, *p =* 0.002; age, *p <* 0.001; sex; *p =* 0.007). (b) Right ICA PI (interaction, *p =* 0.008; age, *p <* 0.001; sex, *p =* 0.005). (c) Left middle cerebral artery (MCA) PI (interaction, *p =* 0.069; age, *p <* 0.001; sex, *p =* 0.335). (d) Right MCA PI (interaction, *p =* 0.304; age, *p <* 0.001; sex, *p =* 0.956). (e) Basilar artery PI (interaction, *p =* 0.492; age, *p <* 0.001; sex, *p =* 0.692). Significance at *p <* 0.05.

Pulsatility in young, middle‐aged and older males and females is displayed in Figure 1. Young females had lower PI compared with young males in both the left ICA (*p =* 0.020) and right ICA (*p <* 0.001) (Figure 1a,b). In addition, older females had greater PI compared with older males in the right ICA (*p =* 0.032), but not in the left ICA (*p =* 0.079). Middle‐aged females had a significantly lower PI compared with middle‐aged males in the right ICA (*p =* 0.008), but not in the left ICA (*p =* 0.119). When assessing PI in the ICAs in males and females with age, there were significant age × sex interactions and main effects of age and sex for PI in both the left (interaction, *p =* 0.002; age, *p <* 0.001; sex, *p =* 0.007) and right ICAs (interaction, *p =* 0.008; age, *p <* 0.001; sex, *p =* 0.005), whereby females had lower PI than males at a young age, similar PI to males around middle‐age and greater PI than males at older age (Figure 2a,b).

### 3.3 MCA pulsatility index

Older females had greater PI compared with older males in the right MCA (*p =* 0.024) but not the left MCA (*p =* 0.119), but there were no differences in PI in the left or right MCAs between males and females in the young (left, *p =* 0.750; right, *p =* 0.178) or middle‐aged (left, *p =* 0.776; right, *p =* 0.526) groups (Figure 1c,d). When assessing PI in the MCAs in males and females with age, there were only main effects of age, but no significant age × sex interactions or main effects of sex, in the left (interaction, *p =* 0.069; age, *p <* 0.001; sex, *p =* 0.335) or right (interaction, *p =* 0.304; age, *p <* 0.001; sex, *p =* 0.956) MCAs. Both males and females had greater PI with increasing age in both MCAs (Figure 2c,d).

### 3.4 Basilar artery pulsatility index

There were no sex differences for PI in the basilar artery between males and females in the young (*p =* 0.690), middle‐aged (*p =* 0.089) or older (*p =* 0.228) groups (Figure 1e). When assessing PI in males and females with age in the basilar artery, there was only a main effect of age, but no significant age × sex interaction or main effect of sex (interaction, *p =* 0.492; age, *p <* 0.001; sex, *p =* 0.692), whereby both males and females had greater PI with increasing age (Figure 2e).

### 3.5 Within‐ICA damping factor

**FIGURE 3:**
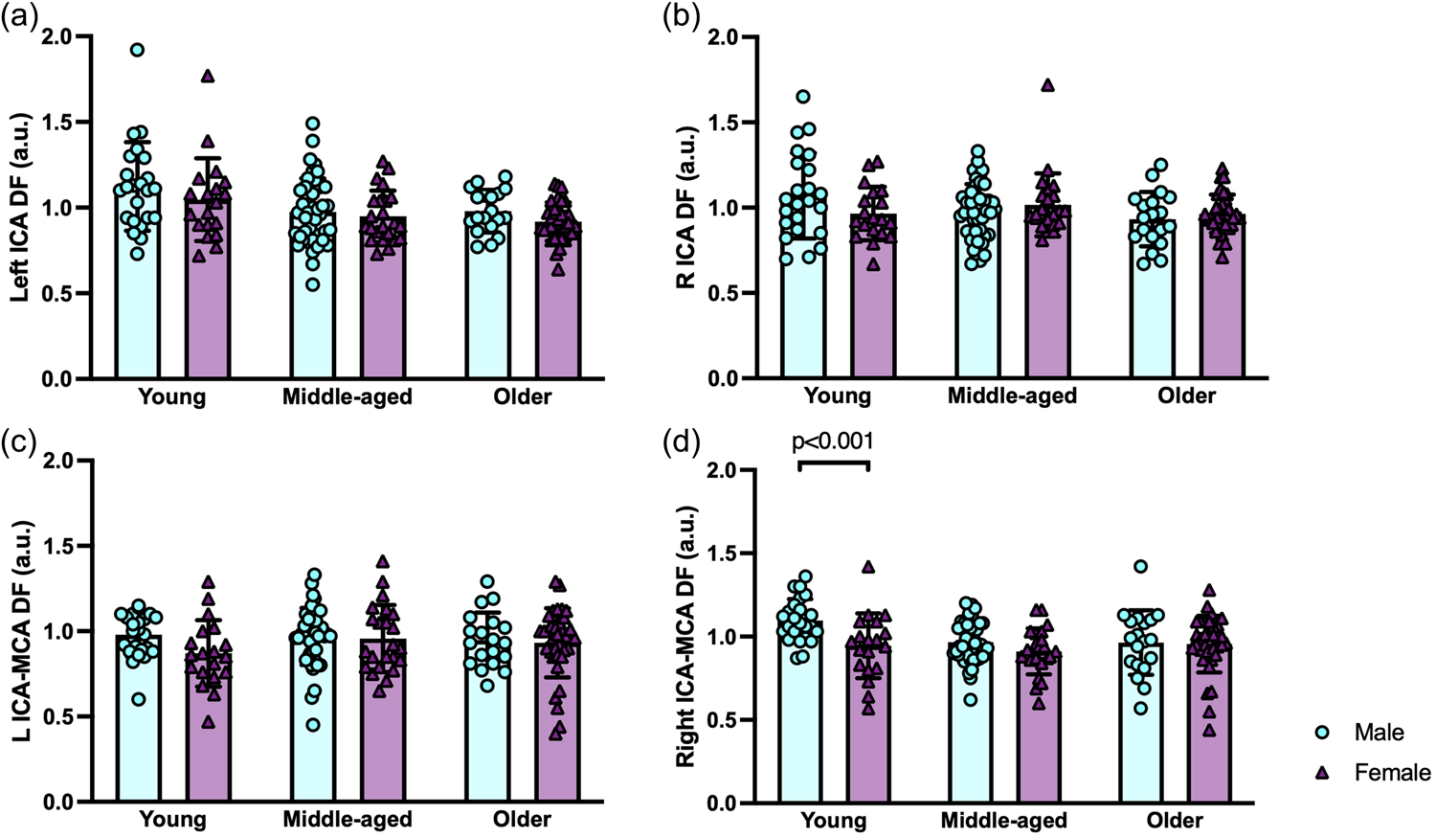
Sex differences in damping factor (DF) for males (turquoise, circles) and females (plum, triangles) in young (20–35 years; 24 males and 18 females), middle‐aged (45–65 years; 43 males and 23 females) and older (70–89 years; 19 males and 35 females) age groups are shown. Data are presented using bar graphs, with error bars indicating SD and overlapping data points shown. (a) Left internal carotid artery (ICA) DF (sex differences within: young, *p =* 1.000; middle‐aged, *p =* 0.291; and older, *p =* 0.084). (b) Right ICA DF (sex differences within: young, *p =* 0.416; middle‐aged, *p =* 0.752; and older, *p =* 0.465). (c) Left ICA to middle cerebral artery (MCA) DF (sex differences within: young, *p =* 0.195; middle‐aged, *p =* 0.561; and older, *p =* 0.751). (d) Right ICA to MCA DF (sex differences within: young, *p <* 0.001; middle‐aged, *p =* 0.107; and older, *p =* 0.964). Differences in DF between males and females within each age group were evaluated using Welch's *t*‐tests or Mann–Whitney *U*‐tests, with significance at *p <* 0.05.

**FIGURE 4:**
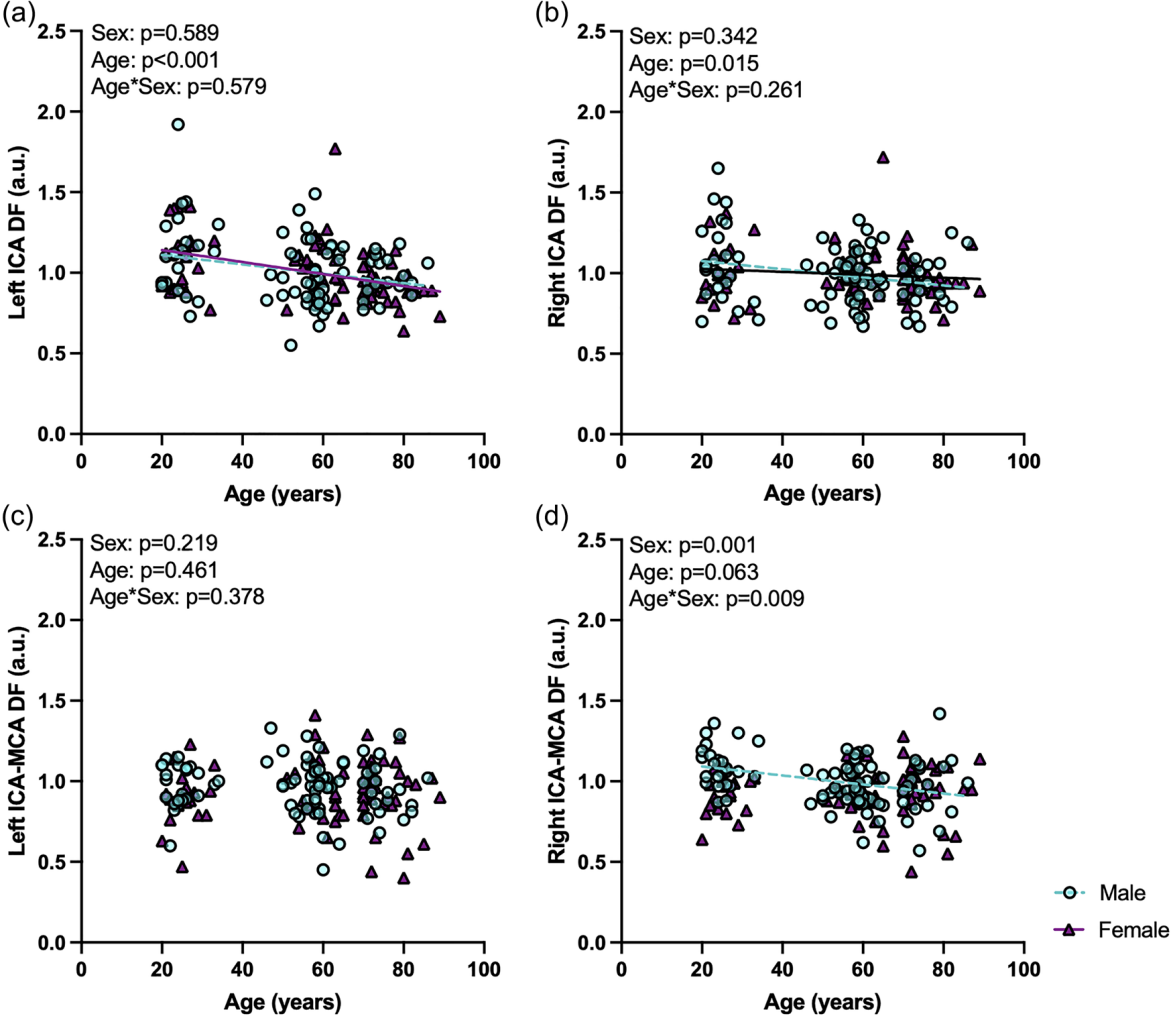
Damping factor (DF) with continuous age and sex was investigated using general linear models or generalized linear models with a gamma distribution as needed in males (*n* = 86; turquoise, circles) and females (*n* = 76; plum, triangles). (a) Left internal carotid artery (ICA) DF (interaction, *p =* 0.579; age, *p <* 0.001; sex, *p =* 0.589). (b) Right ICA DF (interaction, *p =* 0.261; age, *p =* 0.015; sex, *p =* 0.342). (c) Left ICA to middle cerebral artery (MCA) DF (interaction, *p =* 0.378; age, *p =* 0.461; sex, *p =* 0.219). (d) Right ICA to MCA DF (interaction, *p =* 0.009; age, *p =* 0.063; sex, *p =* 0.001). Significance at *p <* 0.05.

There were no sex differences for DF within the left or right ICAs in young (left, *p =* 1.000; right, *p =* 0.416), middle‐aged (left, *p =* 0.291; right, *p =* 0.752) or older (left, *p =* 0.084; right, *p =* 0.465) groups (Figure 3a,b). When assessing DF in males and females with age and within the ICAs, there were no significant age × sex interactions or main effects of sex, but there were main effects of age, within the left (interaction, *p =* 0.579; age, *p <* 0.001; sex, *p =* 0.589) and the right (interaction, *p =* 0.261; age, *p =* 0.015; sex, *p =* 0.342) ICAs (Figure 4a,b).

### 3.6 ICA–MCA damping factor

There were no sex differences in left ICA–MCA DF between males and females in young (*p =* 0.195), middle‐aged (*p =* 0.561) or older (*p =* 0.751) groups (Figure 3c). For right ICA–MCA DF, there were sex differences such that young females had lower DF compared with young males (*p <* 0.001); however, there were no other sex differences in middle‐aged (*p =* 0.107) or older (*p =* 0.964) groups (Figure 3d). When assessing DF in males and females with age and between the ICAs and MCAs, there was not a significant age × sex interaction or main effects of age or sex in DF between the left ICA and MCA (interaction, *p =* 0.378; age, *p =* 0.461; sex, *p =* 0.219), but there was a significant age × sex interaction and main effect of sex on DF between the right ICA and MCA (interaction, *p =* 0.009; age, *p =* 0.063; sex, *p =* 0.001) (Figure 4c,d).

## 4 DISCUSSION

In this study, we examined sex differences in cerebral pulsatility and damping across the adult lifespan in a large cohort and within multiple cerebral arteries using 4D Flow MRI. First, we observed sex differences in cerebral pulsatility (PI) over the lifespan that were artery specific, with differences being more apparent in the ICAs as females had greater age‐related increases in cerebral pulsatility compared with males. Second, we did not observe sex differences in damping (DF) with advancing age, except between the right ICA and MCA, where young females had lower damping than young males. Interesting, there was a sex‐specific decline in damping between the right ICA and MCA, whereby age‐related declines in damping were more present in males. Collectively, these data suggest that age‐related increases in cerebral pulsatility are more apparent in females, with differences being more profound in proximal arteries than in distal arteries, and that the underlying mechanism might not be attributable to changes in damping. These data also suggest that age‐related changes in cerebral damping are unique to either within an artery (i.e. within the ICA) or between arteries (i.e. between the ICA and MCA) but might be region dependent (e.g. left vs. right) and sex specific.

### 4.1 Sex differences in cerebral pulsatility and damping

Our cross‐sectional analysis suggests that females have greater age‐related increases in cerebral pulsatility in comparison to males in multiple cerebral arteries involved in the anterior circulation (ICAs and MCAs) and especially in proximal, or feed, arteries (ICAs). This observation supports our previous work (Alwatban et al., 2021) and that of others (Dempsey et al., 2024; Lefferts et al., 2023), although their findings were either limited to velocity analysis of a single cerebral artery assessed with TCD (unilateral measurement of the MCA) (Alwatban et al., 2021; Lefferts et al., 2023) or did not consider distal arteries (MCAs) in their analysis (Dempsey et al., 2024). In previous studies that used a more comprehensive method to assess cerebral pulsatility in multiple cerebral arteries, findings have been more variable. Zarrinkoob et al. (2016) reported no sex differences in cerebral pulsatility using two‐dimensional phase contrast MRI (2D PC MRI); however, a limitation with their findings is that they collapsed age groups and averaged left and right PI of each artery for the sex difference analyses, which might have masked age‐ and region‐specific differences between sexes. Findings from Van Tuijl et al. (2020), also using 2D PC MRI, reported greater cerebral pulsatility in males compared with females within the left and right ICAs; however, comparisons were made without stratifying for age, which would have masked important differences, such as those we observed between males and females in the young and older age groups in the present study.

Another new finding of our study was that the age‐related decreases in damping were specific to within an artery rather than between two arteries. When assessing damping within the ICA, males and females had lower damping with age. When assessing damping between the ICA and MCA, it was only between the right ICA and MCA that males had lower damping with age. Although interestingly, females had lower damping in comparison to males at a young age between the right ICA and MCA. Our findings suggest that the damping capacity of the ICAs is affected by age more profoundly than between the ICAs and MCAs. These findings are in partial agreement with findings from Alwatban et al. (2021), Lefferts et al. (2023) and Dempsey et al. (2024). However, our reported differences in cerebral pulsatility between females and males contrast with findings from Dempsey et al. (2024), which could be attributable to the smaller sample size of Dempsey et al. (2024). With our larger cohort, we were able to distinguish important sex differences that might otherwise have been difficult to identify. Additionally, incorporating distal arteries allowed us to conduct a more comprehensive analysis and confirm our findings from our past work, which had only data from the MCAs.

When considering the underlying mechanisms associated with the sex differences in cerebral pulsatility and damping, factors such as sex hormones (i.e. oestrogen and progesterone) have been shown to affect vascular function directly. For instance, oestrogen acts as a protective mechanism for the cardiovascular system via oestrogen‐specific receptors (Arnal et al., 2010), and both oestrogen and progesterone can also play a role in cerebrovascular function (Barnes et al., 2019; Iwamoto et al., 2021; Krause et al., 2006; Stirone et al., 2003). In the event of a disturbance in the secretion of sex hormones (e.g. menopause), cardiovascular and cerebrovascular function might be impaired (Barnes et al., 2019; DuPont et al., 2019). Indeed, low circulating levels of oestrogen observed with menopause have been demonstrated to leave females vulnerable to deleterious conditions, such as elevated BP and arterial stiffening (DuPont et al., 2019; Lefferts et al., 2020), which have been associated with increased cerebral pulsatility (Arts et al., 2022; Fico et al., 2022; Lefferts et al., 2020). Furthermore, levels of oestrogen have been shown to affect vasodilatation within the ICAs specifically (Iwamoto et al., 2021). Iwamoto et al. (2021) reported reduced shear‐mediated vasodilatation within the ICAs across the menopausal transition in females, with postmenopausal females demonstrating the greatest reduction in ICA shear‐mediated vasodilatation in response to a vasodilatory stimulus. In our study, although we did not assess cerebral pulsatility and damping in response to a stimulus, we identified a greater age‐related increase in cerebral pulsatility within the ICAs, whereby young premenopausal females had lower cerebral pulsatility compared with young males in both ICAs, and older postmenopausal females had greater cerebral pulsatility compared with older males within the right ICA. In addition, the age‐related decline in cerebral damping within females was only observed within the ICAs, without an observable difference in cerebral damping between the ICAs and MCAs. Taken together, the lower secretion of sex hormones, such as oestrogen, through age‐related events, such as menopause, could put females at greater risk of elevated cerebral pulsatility and decreased damping, especially within the proximal feed arteries, such as the ICAs.

### 4.2 Cerebral pulsatility and damping with age

Our data demonstrated an increase in cerebral pulsatility and decrease in cerebral damping with age, which is consistent with previous literature (Dempsey et al., 2024; Lefferts et al., 2020; Martin et al., 1994; Tarumi et al., 2014; Zarrinkoob et al., 2016). Furthermore, our data show an age‐related increase in cerebral pulsatility within multiple cerebral arteries and an age‐related decrease in cerebral damping unique to within an artery (i.e. within the ICA) or between two arteries (i.e. between the ICA and MCA). Our data also establish sex‐specific trends in the increase in cerebral pulsatility with age. It has been postulated that the age‐related increase in cerebral pulsatility might be attributable, in part, to the increase in aortic stiffness observed with ageing (Webb et al., 2012; Wohlfahrt et al., 2014). Previous work from our laboratory has demonstrated a positive association between arterial stiffness and cerebral pulsatility (Fico et al., 2022). A prevailing theory is that in younger individuals, there is an impedance mismatch between the aorta and the first order central arteries (common carotid arteries), in which there is lower impedance in the compliant aorta and higher impedance in the more resistant common carotid arteries (Mitchell et al., 2011). This impedance mismatch promotes adequate attenuation of the pulsatile energy of the forward wave and creates continuous (non‐pulsatile) flow prior to reaching distal cerebral arteries (Mitchell et al., 2011). As the aorta stiffens and becomes less compliant, aortic impedance increases and lessens the mismatch between the aorta and carotid arterial impedance. This would, in theory, hinder the ability of the reflecting wave to attenuate the forward‐travelling pulsatile energy and allow transmission of pulsatile energy towards downstream cerebral arteries. We were unable to include aortic stiffness measurements in the present study; therefore, further exploration of this hypothesis needs to be conducted.

Our data also suggest a decrease in damping with age, which supports the findings from Zarrinkoob et al. (2016) and Dempsey et al. (2024). Specifically, damping appeared to decrease within the left ICA with advancing age, which was a new finding of our study. It has been reported previously that the ICAs might play a unique role in attenuation of flow pulsatility. First, proximal segments of the ICA pass through the carotid canal, which limits distensibility of the arteries (Van Tuijl et al., 2020). Second, distal segments of the ICA form the carotid siphon, whose tortuous shape might uniquely attenuate pulsatile flow prior to reaching the MCA (Schubert et al., 2011). Taken together, greater transmission of pulsatile energy towards the ICAs might increase the reliance on damping properties of the distal segments. This hypothesis is supported by findings from Arts et al. (2022) that show a positive association between cerebral damping, measured between the proximal segment of the MCA and arteries distal to the MCA using 2D PC MRI, and arterial stiffness in middle‐aged and older healthy adults. With increased stiffening of the proximal or central arteries observed with ageing, damping within the cerebral circulation can act as a compensatory mechanism to prevent excessive flow pulsatility from reaching the cerebral microcirculation (Faraci & Heistad, 1990); however, chronic exposure to increased pulsatile stress might elicit vascular damage and vascular remodelling, thus impairing the damping capacity over time (Van Tuijl et al., 2020).

### 4.3 Strengths and limitations

A novel aspect of this study is the use of 4D flow MRI, which is an advanced MRI technology that assesses blood velocity across the cardiac cycle in 3D (Markl et al., 2012; Miller et al., 2019; Rivera‐Rivera et al., 2016). This enables the simultaneous measurement of multiple vessels and the integration of velocity values to derive blood flow rates. 4D flow MRI also has good test–retest reliability owing to the standardized regions of interest within each artery, reproducibility across multiple sites, and interobserver agreement (Wen et al., 2019). This study confirmed results from our previous work that used TCD to assess sex differences in cerebral pulsatility with age, with a focus on MCA pulsatility from blood velocity rather than flow (Alwatban et al., 2021). Another important strength of this study is the health of the participants. To isolate the effect of age and sex, we excluded participants with a history of stroke, diabetes or cardiovascular, renal/hepatic, haematological, peripheral vascular or neurovascular disease. In addition, we excluded participants diagnosed with hypertension or taking antihypertensive medications, because diagnosed hypertension and the use of antihypertensive medications have been linked with elevated cerebral pulsatility and lower cerebral damping (Van Den Kerkhof et al., 2023; Webb & Werring, 2022). Thus, excluding individuals diagnosed with hypertension or taking antihypertensive medications allowed for us to isolate further the effects of primary ageing on cerebral pulsatility and damping. Other strengths of this study include the stratification of males and females into young, middle‐aged and older age groups to identify potential sex differences that might otherwise be masked, and assessment of bilateral proximal and distal cerebral arteries to expand our understanding of differences in cerebral pulsatility and damping.

Limitations of this study include the use of cross‐sectional analysis to investigate the effects of age. In addition, participants included for this study were otherwise healthy and reported having varying levels of physical activity, hence generalization to certain populations is not possible. Furthermore, we did not have representation of adults between 35 and 45 years of age and did not include perimenopausal females. We included a middle‐age group made up of postmenopausal females, which provides additional insight into when sex‐specific differences arise across the adult lifespan, especially following menopause. Nevertheless, future work should ensure that those between 35 and 45 years of age, in addition to females across the menopausal transition, are adequately studied.

## 5 CONCLUSION

In summary, our new findings were that age‐related increases in cerebral pulsatility, obtained using 4D flow MRI, were greater in females in comparison to males, especially within the ICAs. Furthermore, females had either lower or similar cerebral pulsatility at an early age and greater cerebral pulsatility at an older age in comparison to males. These results suggest that the age‐related increase in cerebral pulsatility might be sex and artery specific. Regarding cerebral damping, the lack of sex differences in damping within the proximal arteries (ICAs) suggests that a different mechanism might underlies the differences in cerebral pulsatility observed between males and females within those same arteries. Future work should explore mechanisms that might drive the sex‐specific trends we observed in cerebral pulsatility with advancing age. Collectively, our findings on normotensive healthy adults highlight the importance of biological sex when assessing age‐related changes in cerebral haemodynamics. Furthermore, our findings demonstrate the necessity for studying multiple arteries within the brain to assess cerebrovascular health, because changes in cerebrovascular haemodynamics might occur earlier in life within proximal cerebral arteries (ICAs) prior to observable changes in distal cerebral arteries (MCAs).

### 5.1 Future directions

Owing to the sex‐specific risks for cardiovascular and cerebrovascular diseases, future studies could investigate whether these sex differences in cerebral haemodynamics impact sex differences in neurodegenerative disease. In addition, it is necessary to understand the mechanisms underlying the evident sex differences in cerebral haemodynamics, and future studies should include exploration into changes with menopausal hormone status. Lastly, future work should include assessments of multiple arteries to create a global view of potential cerebral haemodynamic changes across the lifespan.

## AUTHOR CONTRIBUTIONS

Brandon G. Fico, Sarean Harmoni A. Gaynor‐Metzinger and Jill N. Barnes designed the experiments. Kathleen B. Miller, Adam T. Corkery, Anna J. Howery, Nicole A. Loggie, Andrew G. Pearson and Jill N. Barnes collected the data. Sarean Harmoni A. Gaynor‐Metzinger, Alexander M. Norby, Brandon G. Fico, Kathleen B. Miller, Anna J. Howery, Leonardo A. Rivera‐Rivera, Ryan D. Zea and Jill N. Barnes analysed the data. Sarean Harmoni A. Gaynor‐Metzinger, Alexander M. Norby, Brandon G. Fico, M. Erin Moir, Kathleen B. Miller, Leonardo A. Rivera‐Rivera, Oliver Wieben, Kevin M. Johnson, Sterling C. Johnson, Ryan D. Zea and Jill N. Barnes interpreted the data. Sarean Harmoni A. Gaynor‐Metzinger, Alexander M. Norby, Brandon G. Fico and Jill N. Barnes drafted the manuscript and prepared figures. All authors edited and revised the manuscript. All authors approved the final version of the manuscript and agree to be accountable for all aspects of the work in ensuring that questions related to the accuracy or integrity of any part of the work are appropriately investigated and resolved. All persons designated as authors qualify for authorship, and all those who qualify for authorship are listed.

## CONFLICT OF INTEREST

S.C.J. serves as a consultant to Enigma Biomedical and ALZPath. All other authors declare no conflict of interest.

## Supporting information



Individual participants' physical characteristics and CCP parameter values

## Data Availability

Requests for access to the data included in this report will be considered by Dr Jill N. Barnes (jnbarnes@wisc.edu) and co‐authors, in compliance with institutional data sharing policies.
