# Supplementary material for: Sex differences in cerebral pulsatility and damping: A 4D flow MRI study
Source: Exp Physiol. 2025 Jul 19;111(2):568–79. doi: 10.1113/EP092630 (PMC12857486; doi:10.1113/EP092630)
Supplement: Supplementary file 1 — Individual participants' physical characteristics and CCP parameter values [file EPH-111-568-s001.docx]

**Results**

*Combined Participant Characteristics*

There were no differences in age (p = 0.060) between males and females when age groups were collapsed. However, males had more years of education (p = 0.003), were taller (p < 0.001), and weighed more (p < 0.001) than females. There were no differences in systolic BP (SBP; p = 0.300) or pulse pressure (PP; p = 0.892) between males and females, but males had greater diastolic BP (DBP; p = 0.021), mean arterial pressure (MAP; p = 0.048) and lower HR (p = 0.004) than females (Table S1).

| **Table S1. Combined Participant Characteristics** | | | |
| --- | --- | --- | --- |
|  | Male (n=87) | Female (n=77) | *p* value |
| Age (years) | 52 (19) | 58 (20) | 0.060 |
| Education (years) | 18 (2) | 16 (2) | **0.003** |
| Height (cm) | 178.7 (6.3) | 163.3 (6.7) | **<0.001** |
| Weight (kg) | 80.2 (10.2) | 63.4 (8.8) | **<0.001** |
| SBP (mmHg) | 125 (12) | 122 (19) | 0.300 |
| DBP (mmHg) | 76 (8) | 73 (8) | **0.021** |
| MAP (mmHg) | 92 (9) | 89 (11) | **0.048** |
| PP (mmHg) | 49 (9) | 50 (15) | 0.892 |
| HR (bpm) | 58 (10) | 62 (9) | **0.004** |
| *Data are presented as mean (SD). DBP, diastolic blood pressure; HR, heart rate; MAP, mean arterial pressure; PP, pulse pressure; SBP, systolic blood pressure. p values indicate differences between males and females within each age group. p < 0.05 are bolded.* | | | |

*Cerebral Pulsatility with Age and Sex and with Pulse Pressure (PP) as a Covariate*

Pulsatility index (PI) was assessed in males and females within the internal carotid arteries (ICAs), middle cerebral arteries (MCAs), and the basilar artery with age and PP as covariates using generalized linear models with a gamma distribution. When assessing PI in the ICAs in males and females with age and with PP as a covariate, there were significant age*sex interactions and main effects of age and sex, but not PP, in both the left (interaction: p = 0.004; age: p < 0.001; sex: p = 0.015; PP: p = 0.369) and the right (interaction: p < 0.001; age: p < 0.001; sex: p = 0.001; PP: p = 0.333) ICAs.

When assessing PI in the middle cerebral arteries (MCAs) in males and females with age and with PP as a covariate, there was not a significant age*sex interaction, but there were main effects of age, sex and PP, in the left MCA (interaction: p = 0.420; age: p < 0.001; sex: p = 0.956; PP: p < 0.001), whereas there was not a age*sex interaction nor main effects of sex and PP, but a significant main effect of age, in the right MCA (interaction: p = 0.591; age: p < 0.001; sex: p = 0.702; PP: p = 0.055).

For basilar PI in males and females with age and PP, there was not a significant age*sex interaction nor main effects of sex and PP, but there was a main effect of age (interaction: p = 0.537; age: p < 0.001; sex: p = 0.732; PP: p = 0. 848).

*Cerebral Damping with Age and Sex and with Pulse Pressure (PP) as a Covariate*

Damping factor (DF) was assessed in males and females within the ICAs as well as between the ICAs and MCAs with age and PP as covariates using general linear models. For DF within the ICAs, there were no significant age*sex interactions nor main effects of sex and PP, but there were significant main effects of age, within both the left (interaction: p = 0.718; age: p < 0.001; sex: p = 0.720; PP: p = 0.458) and right (interaction: p = 0.176; age: p = 0.019; sex: p = 0.240; PP: p = 0.270) ICAs.

When assessing DF between the ICAs and MCAs, there was not a significant age*sex interaction nor main effects of age and sex, but a significant main effect of PP, between the left ICA and MCA (interaction: p = 0.135; age: p = 0.912; sex: p = 0.071; PP: p < 0.001). Whereas, there was a significant age*sex interaction as well as main effect of sex, but no main effects of age and PP, between the right ICA and MCA (interaction: p = 0.002; age: p = 0.079; sex: p < 0.001; PP: p = 0.089).
